# *N*′-[(*E*)-3-Indol-3-ylmethyl­ene]isonicotino­hydrazide monohydrate

**DOI:** 10.1107/S1600536809027329

**Published:** 2009-07-18

**Authors:** Liang-You Xia, Wen-Long Wang, Shan-Heng Wang, Yan-Lan Huang, Shang Shan

**Affiliations:** a Department of Chemistry, Zunyi Normal College, People’s Republic of China; b College of Chemical Engineering and Materials Science, Zhejiang University of Technology, People’s Republic of China

## Abstract

Crystals of the title compound, C_15_H_12_N_4_O·H_2_O, were obtained from a condensation reaction of isonicotinylhydrazine and 3-indolylformaldehyde. The mol­ecule assumes an *E* configuration, with the isonicotinoylhydrazine and indole units located on the opposite sites of the C=N double bond. In the mol­ecular structure the pyridine ring is twisted with respect to the indole ring system, forming a dihedral angle of 44.72 (7)°. Extensive classical N—H⋯N, N—H⋯O, O—H⋯O and O—H⋯N hydrogen bonding and weak C—H⋯O inter­actions are present in the crystal structure.

## Related literature

For the applications of hydrazone derivatives in biology, see: Okabe *et al.* (1993 ▶). For general background to this work, see: Shan *et al.* (2003 ▶); Qiang *et al.* (2007 ▶). For the corresponding (*E*)-3-indolylformaldehyde isonicotinoylhydrazone methanol solvate, see: Tai *et al.* (2003 ▶), and (*E*)-3-indolylformaldehyde isonicotinoylhydrazone ethanol solvate, see: Jing *et al.* (2006 ▶).
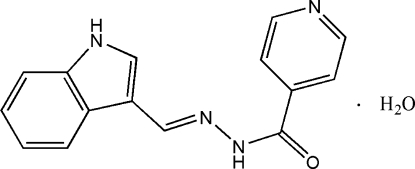


## Experimental

### 

#### Crystal data

C_15_H_12_N_4_O·H_2_O
*M* *_r_* = 282.30
Monoclinic, 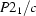

*a* = 7.1984 (11) Å
*b* = 25.327 (4) Å
*c* = 7.9811 (16) Å
β = 104.062 (12)°
*V* = 1411.5 (4) Å^3^
*Z* = 4
Mo *K*α radiation
μ = 0.09 mm^−1^
*T* = 294 K
0.40 × 0.32 × 0.28 mm

#### Data collection

Rigaku R-AXIS RAPID IP diffractometer
Absorption correction: none
9235 measured reflections
2504 independent reflections
1575 reflections with *I* > 2σ(*I*)
*R*_int_ = 0.048

#### Refinement

*R*[*F* ^2^ > 2σ(*F* ^2^)] = 0.055
*wR*(*F* ^2^) = 0.161
*S* = 1.07
2504 reflections
191 parameters
H-atom parameters constrained
Δρ_max_ = 0.18 e Å^−3^
Δρ_min_ = −0.20 e Å^−3^

### 

Data collection: *PROCESS-AUTO* (Rigaku, 1998 ▶); cell refinement: *PROCESS-AUTO*; data reduction: *CrystalStructure* (Rigaku/MSC, 2002 ▶); program(s) used to solve structure: *SIR92* (Altomare *et al.*, 1993 ▶); program(s) used to refine structure: *SHELXL97* (Sheldrick, 2008 ▶); molecular graphics: *ORTEP-3 for Windows* (Farrugia, 1997 ▶); software used to prepare material for publication: *WinGX* (Farrugia, 1999 ▶).

## Supplementary Material

Crystal structure: contains datablocks I, global. DOI: 10.1107/S1600536809027329/xu2554sup1.cif

Structure factors: contains datablocks I. DOI: 10.1107/S1600536809027329/xu2554Isup2.hkl

Additional supplementary materials:  crystallographic information; 3D view; checkCIF report

## Figures and Tables

**Table 1:** Hydrogen-bond geometry (Å, °)

*D*—H⋯*A*	*D*—H	H⋯*A*	*D*⋯*A*	*D*—H⋯*A*
N1—H1*N*⋯N4^i^	0.91	2.06	2.961 (3)	171
N3—H3*N*⋯O1*W*^ii^	0.89	2.11	2.939 (3)	155
O1*W*—H1*A*⋯O1^iii^	0.91	1.90	2.800 (3)	168
O1*W*—H2*A*⋯N2	0.89	2.40	3.223 (3)	152
C12—H12⋯O1*W*^ii^	0.93	2.58	3.466 (3)	159

Symmetry codes: (i) 
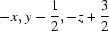
; (ii) 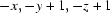; (iii) 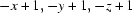.

**Fig. 1.**
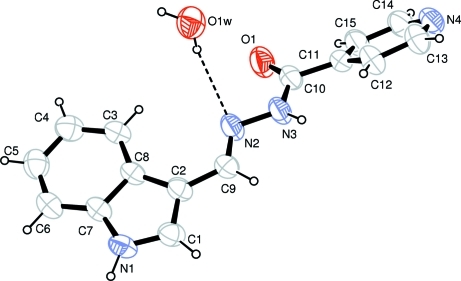
The molecular structure of the title compound with 40% probability displacement (arbitrary spheres for H atoms). Dashed line indicates the hydrogen bonding.

## supplementary crystallographic information

### Comment

The hydrazone derivatives has attracted our much attention because they have shown to be potential DNA damaging and mutagenic agents (Okabe *et al.*, 1993). As part of the ongoing investigation on the relationship between structure and property of hydrazone derivatives (Shan *et al.*, 2003; Qiang *et al.*, 2007) the title compound has recently been prepared in our laboratory and its crystal structure is reported here.

The molecular structure of the title compound is shown in Fig. 1. The pyridine ring is twisted with respect to the indole ring system by a dihedral angle of 44.72 (7)°. The N2—C9 bond distance of 1.293 (3) Å shows a typical C═N double bond. The isonicotinoylhydrazine and indole moieties are located on the opposite sites of the CN bond, thus the molecule assumes an E configuration, which agrees with those found in ethanol solvate compound (Jing *et al.*, 2006) and methanol solvate compound (Tai *et al.*, 2003).

The extensive classic hydrogen bonding and weak C—H···O hydrogen bonding are present in the crystal structure (Table 1).

### Experimental

Isonicotinylhydrazine (1.37 g, 0.01 mol) was dissolved in ethanol (50 ml), the solution was heated at about 333 K for several minutes until the solution cleared. An ethanol solution (20 ml) containing 3-indolylformaldehyde (1.45 g, 0.01 mol) was dropped slowly into the above solution with continuous stirring, and the mixture solution was refluxed for 1.5 h. When the solution had cooled to room temperature, colorless powder crystals appeared. The powder crystals were separated from the solution and washed with cold water three times. Recrystallization was performed twice with a mixture solvent of 2-propanol-water (1:1 *v*/*v*), to obtain single crystals of the title compound.

### Refinement

Water and imino H atoms were located in a difference Fourier map and were refined as riding in as-found relative positions, *U*_iso_(H) = 1.5*U*_eq_(N,*O*). Methyl H atoms were placed in calculated positions with C—H = 0.96 Å and torsion angle was refined to fit the electron density, *U*_iso_(H) = 1.5*U*_eq_(C). Other H atoms were placed in calculated positions with C—H = 0.93 Å, and refined in riding mode with *U*_iso_(H) = 1.2*U*_eq_(C).

### Crystal data

**Table:**

C_15_H_12_N_4_O·H_2_O	*F*(000) = 592
*M**_r_* = 282.30	*D*_x_ = 1.328 Mg m^−^^3^
Monoclinic, *P*2_1_/*c*	Mo *K*α radiation, λ = 0.71073 Å
Hall symbol: -P 2ybc	Cell parameters from 3236 reflections
*a* = 7.1984 (11) Å	θ = 2.8–25.0°
*b* = 25.327 (4) Å	µ = 0.09 mm^−^^1^
*c* = 7.9811 (16) Å	*T* = 294 K
β = 104.062 (12)°	Prism, colorless
*V* = 1411.5 (4) Å^3^	0.40 × 0.32 × 0.28 mm
*Z* = 4	

### Data collection

**Table:**

Rigaku R-AXIS RAPID IP diffractometer	1575 reflections with *I* > 2σ(*I*)
Radiation source: fine-focus sealed tube	*R*_int_ = 0.048
graphite	θ_max_ = 25.2°, θ_min_ = 2.8°
Detector resolution: 10.0 pixels mm^-1^	*h* = −8→8
ω scans	*k* = −30→28
9235 measured reflections	*l* = −9→9
2504 independent reflections	

### Refinement

**Table:**

Refinement on *F*^2^	Secondary atom site location: difference Fourier map
Least-squares matrix: full	Hydrogen site location: inferred from neighbouring sites
*R*[*F*^2^ > 2σ(*F*^2^)] = 0.055	H-atom parameters constrained
*wR*(*F*^2^) = 0.161	*w* = 1/[σ^2^(*F*_o_^2^) + (0.0753*P*)^2^] where *P* = (*F*_o_^2^ + 2*F*_c_^2^)/3
*S* = 1.07	(Δ/σ)_max_ < 0.001
2504 reflections	Δρ_max_ = 0.18 e Å^−^^3^
191 parameters	Δρ_min_ = −0.20 e Å^−^^3^
0 restraints	Extinction correction: *SHELXL97* (Sheldrick, 2008), Fc^*^=kFc[1+0.001xFc^2^λ^3^/sin(2θ)]^-1/4^
Primary atom site location: structure-invariant direct methods	Extinction coefficient: 0.022 (4)

### Special details

**Table:**

Geometry. All e.s.d.'s (except the e.s.d. in the dihedral angle between two l.s. planes) are estimated using the full covariance matrix. The cell e.s.d.'s are taken into account individually in the estimation of e.s.d.'s in distances, angles and torsion angles; correlations between e.s.d.'s in cell parameters are only used when they are defined by crystal symmetry. An approximate (isotropic) treatment of cell e.s.d.'s is used for estimating e.s.d.'s involving l.s. planes.
Refinement. Refinement of *F*^2^ against ALL reflections. The weighted *R*-factor *wR* and goodness of fit *S* are based on *F*^2^, conventional *R*-factors *R* are based on *F*, with *F* set to zero for negative *F*^2^. The threshold expression of *F*^2^ > σ(*F*^2^) is used only for calculating *R*-factors(gt) *etc*. and is not relevant to the choice of reflections for refinement. *R*-factors based on *F*^2^ are statistically about twice as large as those based on *F*, and *R*- factors based on ALL data will be even larger.

### Fractional atomic coordinates and isotropic or equivalent isotropic displacement parameters (Å^2^)

**Table:**

	*x*	*y*	*z*	*U*_iso_*/*U*_eq_	
N1	0.0721 (3)	0.27145 (8)	0.4661 (3)	0.0532 (6)	
H1N	0.0189	0.2388	0.4518	0.080*	
N2	0.2051 (3)	0.44265 (8)	0.6709 (3)	0.0557 (6)	
N3	0.1479 (3)	0.48641 (8)	0.7552 (3)	0.0567 (6)	
H3N	0.0249	0.4904	0.7553	0.085*	
N4	0.0958 (3)	0.66448 (8)	1.0414 (3)	0.0585 (6)	
O1	0.4458 (3)	0.52331 (7)	0.8055 (3)	0.0778 (7)	
O1W	0.2725 (3)	0.51295 (8)	0.3543 (3)	0.0717 (6)	
H1A	0.3680	0.5059	0.3000	0.108*	
H2A	0.2989	0.4927	0.4490	0.108*	
C1	0.0052 (4)	0.31058 (10)	0.5519 (3)	0.0524 (7)	
H1	−0.1042	0.3081	0.5940	0.063*	
C2	0.1222 (4)	0.35481 (10)	0.5685 (3)	0.0485 (7)	
C3	0.4346 (4)	0.36807 (10)	0.4582 (3)	0.0520 (7)	
H3	0.4625	0.4023	0.4989	0.062*	
C4	0.5486 (4)	0.34271 (11)	0.3671 (4)	0.0603 (8)	
H4	0.6518	0.3606	0.3429	0.072*	
C5	0.5118 (4)	0.29032 (12)	0.3099 (4)	0.0630 (8)	
H5	0.5931	0.2741	0.2511	0.076*	
C6	0.3582 (4)	0.26257 (10)	0.3389 (3)	0.0546 (7)	
H6	0.3348	0.2278	0.3019	0.066*	
C7	0.2390 (4)	0.28874 (9)	0.4262 (3)	0.0469 (6)	
C8	0.2756 (3)	0.34103 (9)	0.4881 (3)	0.0444 (6)	
C9	0.0874 (4)	0.40330 (10)	0.6509 (3)	0.0511 (7)	
H9	−0.0232	0.4063	0.6909	0.061*	
C10	0.2762 (4)	0.52496 (10)	0.8150 (4)	0.0539 (7)	
C11	0.2053 (4)	0.57134 (9)	0.8990 (3)	0.0483 (7)	
C12	0.0156 (4)	0.58804 (10)	0.8584 (3)	0.0587 (8)	
H12	−0.0785	0.5685	0.7835	0.070*	
C13	−0.0314 (4)	0.63452 (10)	0.9316 (4)	0.0611 (8)	
H13	−0.1585	0.6454	0.9026	0.073*	
C14	0.2765 (4)	0.64680 (11)	1.0837 (4)	0.0629 (8)	
H14	0.3666	0.6662	1.1632	0.076*	
C15	0.3374 (4)	0.60145 (10)	1.0164 (4)	0.0590 (8)	
H15	0.4652	0.5913	1.0493	0.071*	

### Atomic displacement parameters (Å^2^)

**Table:**

	*U*^11^	*U*^22^	*U*^33^	*U*^12^	*U*^13^	*U*^23^
N1	0.0506 (13)	0.0365 (12)	0.0698 (15)	−0.0060 (10)	0.0094 (11)	−0.0002 (11)
N2	0.0574 (14)	0.0399 (13)	0.0711 (15)	0.0061 (11)	0.0184 (12)	−0.0088 (11)
N3	0.0554 (13)	0.0399 (13)	0.0771 (16)	0.0050 (11)	0.0207 (12)	−0.0113 (11)
N4	0.0716 (16)	0.0416 (13)	0.0618 (14)	0.0087 (12)	0.0153 (12)	−0.0015 (11)
O1	0.0638 (14)	0.0592 (13)	0.1204 (18)	−0.0004 (11)	0.0418 (13)	−0.0204 (12)
O1W	0.0577 (12)	0.0756 (14)	0.0845 (14)	0.0109 (10)	0.0221 (11)	−0.0006 (11)
C1	0.0469 (15)	0.0442 (15)	0.0663 (17)	0.0017 (13)	0.0139 (13)	0.0062 (13)
C2	0.0488 (15)	0.0374 (14)	0.0575 (16)	0.0058 (12)	0.0093 (13)	0.0040 (12)
C3	0.0542 (16)	0.0382 (14)	0.0616 (17)	−0.0011 (13)	0.0099 (14)	0.0032 (12)
C4	0.0524 (16)	0.0555 (18)	0.0711 (19)	0.0016 (14)	0.0111 (15)	0.0059 (15)
C5	0.0606 (18)	0.0628 (18)	0.0662 (19)	0.0130 (16)	0.0165 (15)	−0.0018 (15)
C6	0.0597 (17)	0.0408 (15)	0.0580 (17)	0.0048 (13)	0.0039 (14)	−0.0042 (12)
C7	0.0464 (15)	0.0369 (14)	0.0524 (15)	0.0020 (12)	0.0021 (12)	−0.0006 (12)
C8	0.0454 (14)	0.0339 (14)	0.0491 (14)	0.0025 (12)	0.0021 (12)	0.0007 (11)
C9	0.0558 (16)	0.0411 (15)	0.0587 (16)	0.0054 (13)	0.0185 (13)	0.0041 (12)
C10	0.0569 (18)	0.0396 (15)	0.0677 (18)	0.0069 (13)	0.0203 (14)	0.0001 (13)
C11	0.0552 (16)	0.0371 (14)	0.0564 (16)	0.0041 (12)	0.0206 (13)	0.0036 (12)
C12	0.0615 (18)	0.0461 (15)	0.0633 (17)	0.0100 (14)	0.0048 (14)	−0.0080 (13)
C13	0.0632 (18)	0.0543 (17)	0.0625 (18)	0.0154 (15)	0.0088 (15)	−0.0032 (14)
C14	0.0634 (19)	0.0478 (17)	0.076 (2)	−0.0035 (15)	0.0131 (16)	−0.0070 (14)
C15	0.0500 (16)	0.0466 (16)	0.081 (2)	0.0017 (13)	0.0165 (15)	−0.0072 (14)

### Geometric parameters (Å, °)

**Table:**

N1—C1	1.358 (3)	C3—H3	0.9300
N1—C7	1.387 (3)	C4—C5	1.407 (4)
N1—H1N	0.9057	C4—H4	0.9300
N2—C9	1.293 (3)	C5—C6	1.376 (4)
N2—N3	1.409 (3)	C5—H5	0.9300
N3—C10	1.349 (3)	C6—C7	1.397 (3)
N3—H3N	0.8916	C6—H6	0.9300
N4—C13	1.338 (3)	C7—C8	1.416 (3)
N4—C14	1.339 (3)	C9—H9	0.9300
O1—C10	1.242 (3)	C10—C11	1.502 (3)
O1W—H1A	0.9147	C11—C15	1.389 (3)
O1W—H2A	0.8945	C11—C12	1.391 (4)
C1—C2	1.388 (3)	C12—C13	1.392 (3)
C1—H1	0.9300	C12—H12	0.9300
C2—C9	1.444 (3)	C13—H13	0.9300
C2—C8	1.449 (3)	C14—C15	1.384 (4)
C3—C4	1.380 (4)	C14—H14	0.9300
C3—C8	1.403 (3)	C15—H15	0.9300
			
C1—N1—C7	108.6 (2)	N1—C7—C6	129.5 (2)
C1—N1—H1N	122.6	N1—C7—C8	108.3 (2)
C7—N1—H1N	128.5	C6—C7—C8	122.2 (2)
C9—N2—N3	114.0 (2)	C3—C8—C7	119.2 (2)
C10—N3—N2	118.9 (2)	C3—C8—C2	134.4 (2)
C10—N3—H3N	120.8	C7—C8—C2	106.4 (2)
N2—N3—H3N	119.7	N2—C9—C2	122.1 (2)
C13—N4—C14	116.3 (2)	N2—C9—H9	119.0
H1A—O1W—H2A	105.0	C2—C9—H9	119.0
N1—C1—C2	110.9 (2)	O1—C10—N3	123.6 (2)
N1—C1—H1	124.6	O1—C10—C11	119.9 (2)
C2—C1—H1	124.6	N3—C10—C11	116.5 (2)
C1—C2—C9	124.2 (2)	C15—C11—C12	117.6 (2)
C1—C2—C8	105.9 (2)	C15—C11—C10	118.6 (2)
C9—C2—C8	130.0 (2)	C12—C11—C10	123.7 (2)
C4—C3—C8	118.4 (2)	C11—C12—C13	119.0 (3)
C4—C3—H3	120.8	C11—C12—H12	120.5
C8—C3—H3	120.8	C13—C12—H12	120.5
C3—C4—C5	121.4 (3)	N4—C13—C12	123.9 (3)
C3—C4—H4	119.3	N4—C13—H13	118.1
C5—C4—H4	119.3	C12—C13—H13	118.1
C6—C5—C4	121.5 (3)	N4—C14—C15	124.0 (3)
C6—C5—H5	119.3	N4—C14—H14	118.0
C4—C5—H5	119.3	C15—C14—H14	118.0
C5—C6—C7	117.2 (2)	C14—C15—C11	119.2 (3)
C5—C6—H6	121.4	C14—C15—H15	120.4
C7—C6—H6	121.4	C11—C15—H15	120.4

### Hydrogen-bond geometry (Å, °)

**Table:**

*D*—H···*A*	*D*—H	H···*A*	*D*···*A*	*D*—H···*A*
N1—H1N···N4^i^	0.91	2.06	2.961 (3)	171
N3—H3N···O1W^ii^	0.89	2.11	2.939 (3)	155
O1W—H1A···O1^iii^	0.91	1.90	2.800 (3)	168
O1W—H2A···N2	0.89	2.40	3.223 (3)	152
C12—H12···O1W^ii^	0.93	2.58	3.466 (3)	159

Symmetry codes: (i) −*x*, *y*−1/2, −*z*+3/2; (ii) −*x*, −*y*+1, −*z*+1; (iii) −*x*+1, −*y*+1, −*z*+1.
